# Supplementary material for: The computational and neural substrates of moral strategies in social decision-making
Source: Nat Commun. 2019 Apr 2;10:1483. doi: 10.1038/s41467-019-09161-6 (PMC6445121; doi:10.1038/s41467-019-09161-6)
Supplement: Supplementary file 1 — Supplementary information [file 41467_2019_9161_MOESM1_ESM.pdf]

Supplementary information

The computational and neural substrates of moral strategies in social decision-making

Van Baar et al.

Supplementary Table 1

		All	GA	GR	IA	MO	Group differences	
	count	57	5	7	24	21	*statistic*	*value*
Gender	F	39	2	5	17	15	$X^2(3)$	2.05
	M	18	3	2	7	6	*p*	0.56
Age	Mean	21.3	20.4	20.4	21.1	22.0	*F(3,53)*	1.65
	s.d.	2.1	1.5	2.4	1.7	2.4	*p*	0.19
SVO	Mean	33.4	33.6	25.1	38.0	30.8	*F(3,53)*	5.84
	s.d.	9.0	12.1	10.9	5.1	8.7	*p*	0.002**
GI: Moral standards	Mean	43.7	47.4	42.4	44.2	42.7	*F(3,53)*	0.98
	s.d.	6.1	7.1	4.0	6.8	5.5	*p*	0.41
GI: State guilt	Mean	24.4	23.8	22.1	25.1	24.6	*F(3,53)*	0.37
	s.d.	6.6	6.6	6.8	7.5	5.7	*p*	0.77
GI: Trait guilt	Mean	49.6	47.8	42.9	50.9	50.7	*F(3,53)*	1.23
	s.d.	10.5	11.0	10.8	10.4	10.3	*p*	0.31
Model error (sum of squares)	Mean	315.9	364.2	265.4	298.9	340.6	*F(3,53)*	0.68
	s.d.	153.8	123.4	202.5	117.0	180.9	*p*	0.57
Theta	Mean	0.15	0.14	0.4	0.06	0.17		
	s.d.	0.13	0.05	0.07	0.05	0.08		
Phi	Mean	-0.01	-0.07	-0.02	0.04	-0.05		
	s.d.	0.07	0.02	0.09	0.03	0.05		

*Descriptive statistics of individual difference measures, as well as modeling results, in the total sample ('All') and split by moral strategy group (GA, GR, IA, MO). Test statistics of differences between the groups are presented on the right of the table. ** p < 0.01.*

Supplementary Figure 1

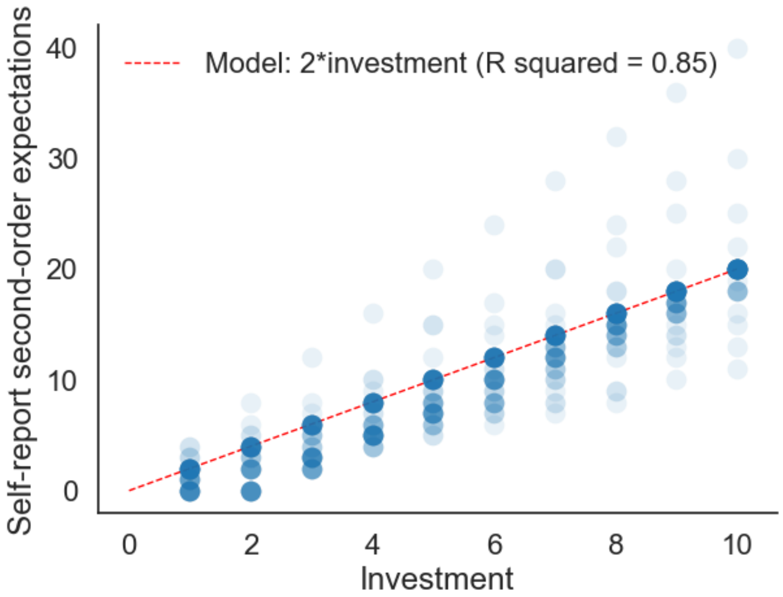

Model for second-order expectations. In the guilt term of the guilt aversion model and the Moral Strategy Model, second-order expectations (Trustee's belief about Investor's expectations) were kept equal across participants and set to half the amount the Investor believes the Trustee has (i.e. $E_2(E_1(S_2)) = ½ * E_1(M) * I$*). Self-report data plotted here confirm that this is a good representation of the true second-order expectations of the Trustees. Note that since self-report was elicited for all investment levels at once, after the scanner experiment, these data points may be a noisy representation of the true second-order expectations during the Hidden Multiplier Trust Game.*

Supplementary Figure 2

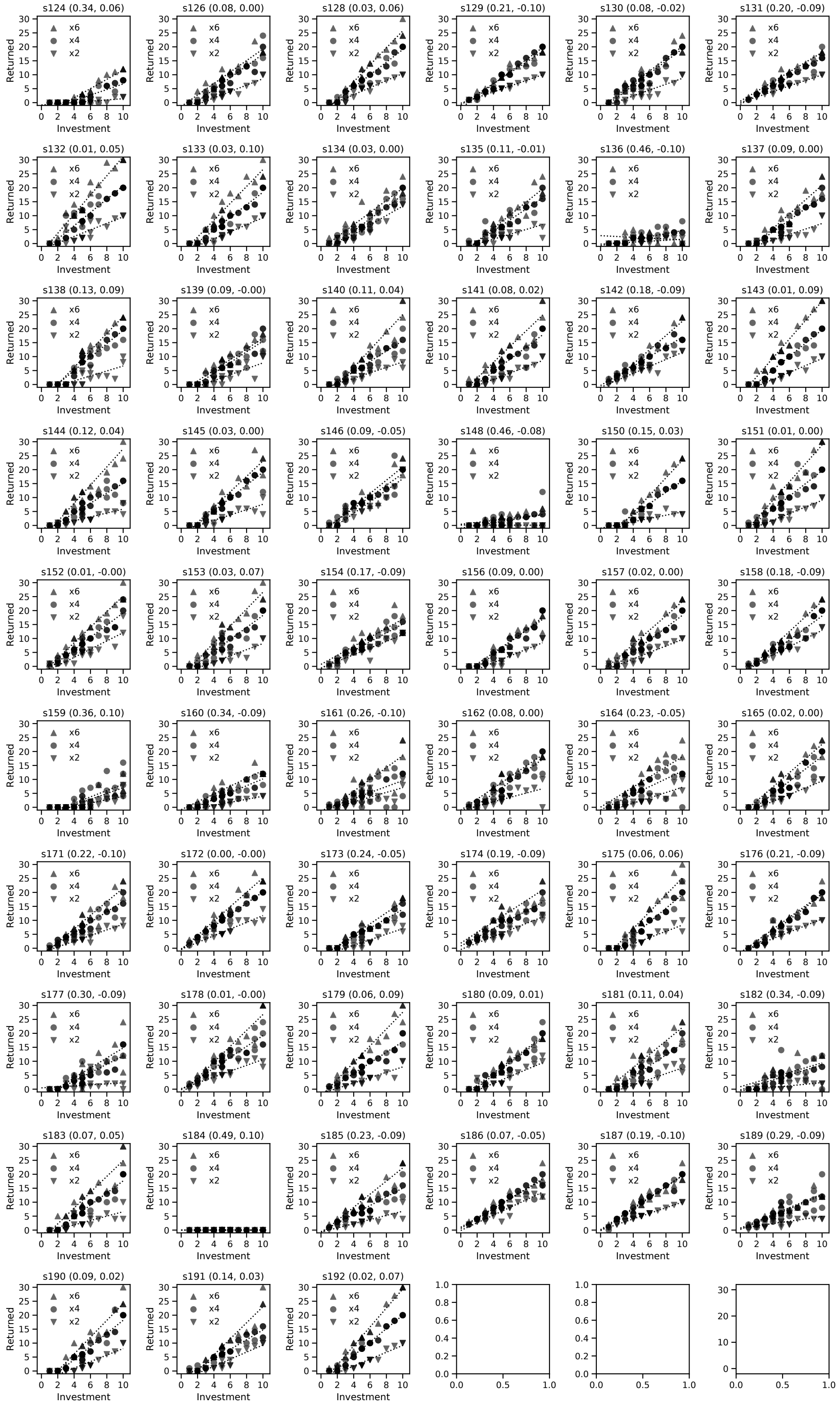

All participants' task behavior. Participant numbers are written after 's' in axis title, followed by the pair of best-fitting model parameters (theta, phi).

Supplementary figure 3

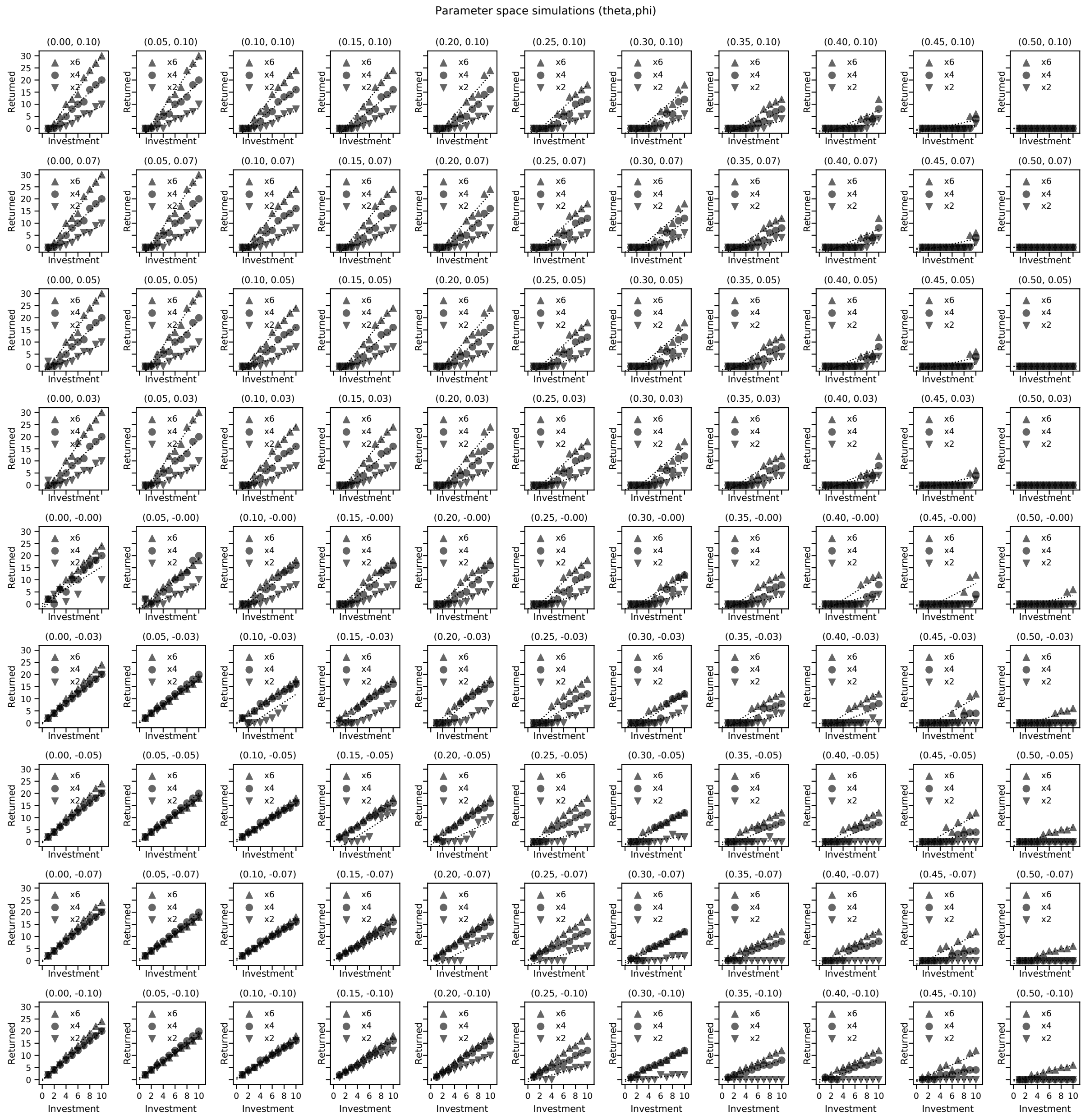

Simulations of the Moral Strategy model in the Hidden Multiplier Trust Game. Simulations generated with theta and phi between the parameter bounds $0 \leq \Theta \leq 0.5$ and $-0.1 \leq \Phi \leq 0.1$.

Supplementary Figure 4

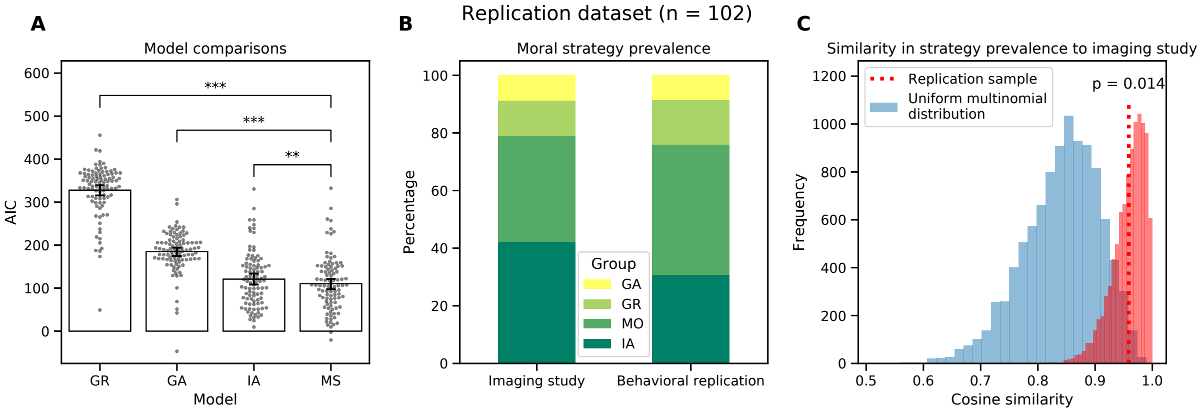

Replication experiment. We conducted a direct behavioral replication of the Hidden-Multiplier Trust Game experiment with n *= 102 (manuscript in preparation). A) Model comparisons on these replication data show that the Moral Strategy model was again the best model to describe the observed behavior.* P*-values from one-sample t-tests on AIC difference scores: **** $p < 0.001$*; *** $p < 0.01$*. B) The distribution of the four moral strategies in this replication sample, side-by-side with the distribution obtained in the imaging study described in this paper. A chi-square test does not reject the null hypothesis that the two distributions are the same:* $X^2(3) = 2.22$, $p = 0.53$*. C) Moreover, the similarity in strategy prevalence between the two experiments is much greater than one would expect to obtain by chance alone (bootstrapped mean cosine similarity* $r = 0.96$*, permutation test* $p = 0.014$*). See Methods for more details on this replication study.*

Supplementary Figure 5

A

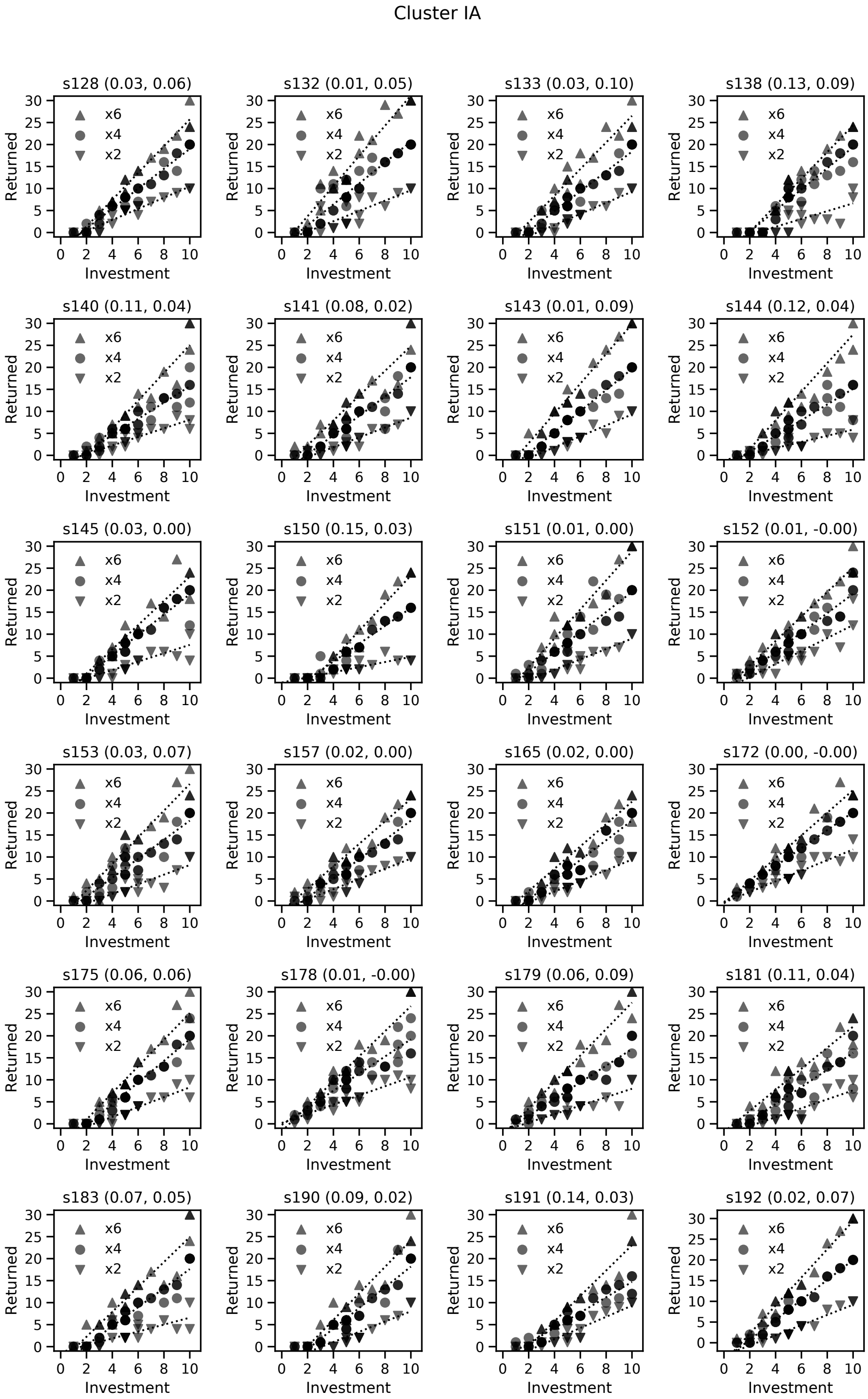

Task behavior of each participant in the inequity-averse (IA) group.

B

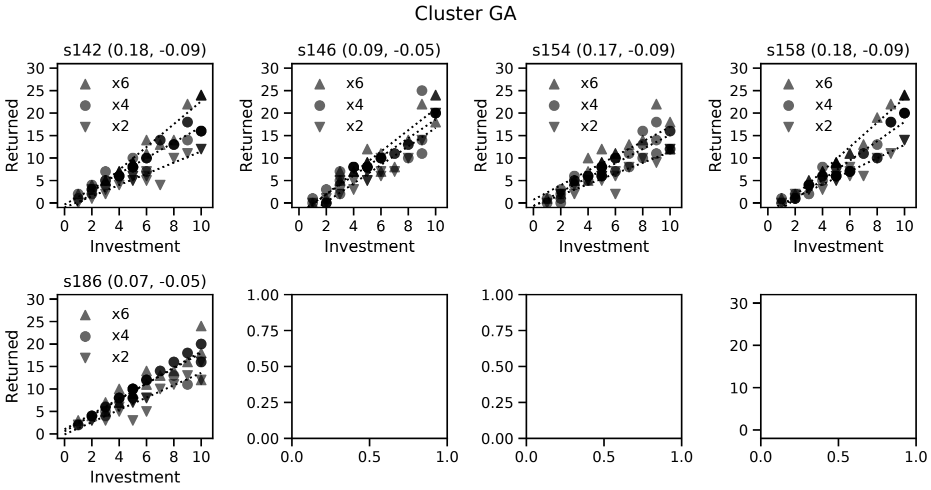

Task behavior of each participant in the guilt-averse (GA) group.

C

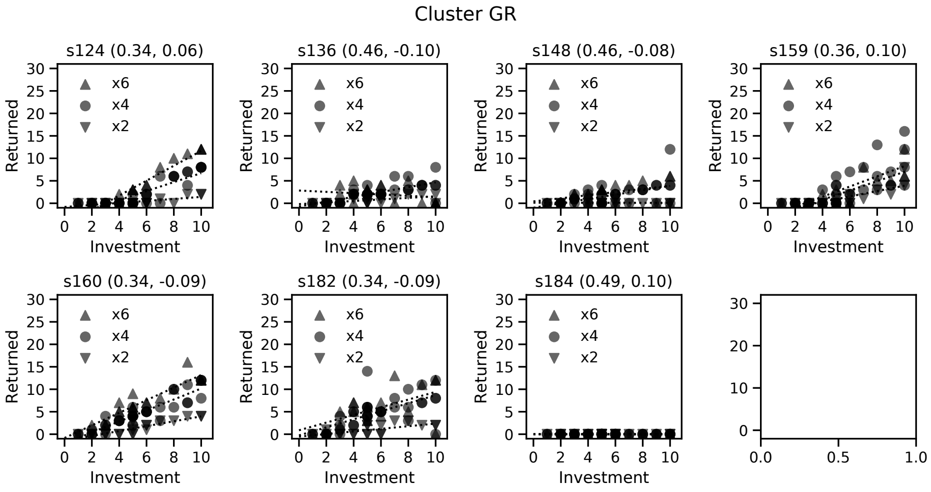

Task behavior of each participant in the greedy (GR) group.

D

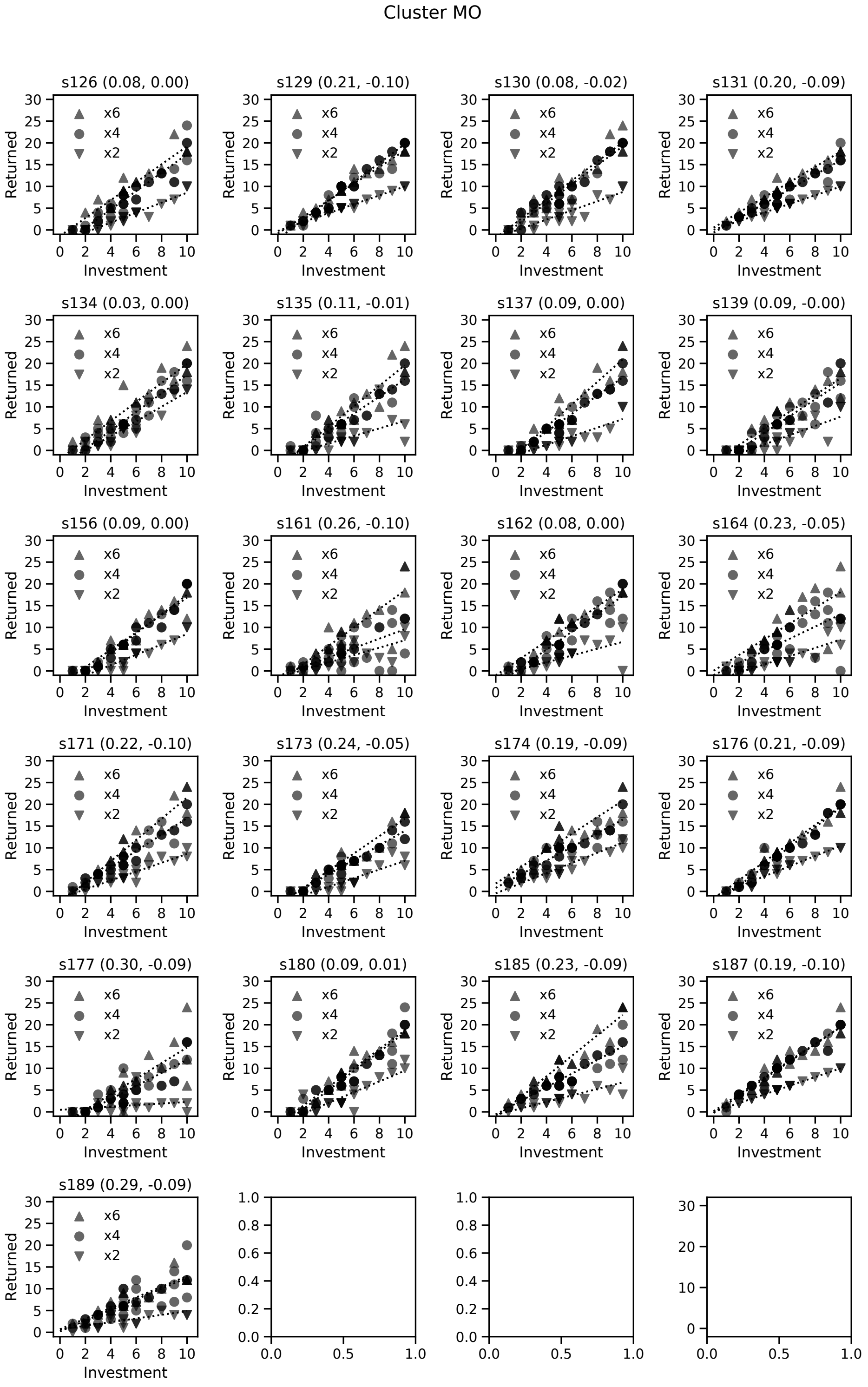

Task behavior of each participant in the morally opportunistic (MO) group.

Supplementary Figure 6

A

Task behavior over time. Each participant in the inequity-averse (IA) group, split by early, middle, and late trials to illustrate choice consistency over time.

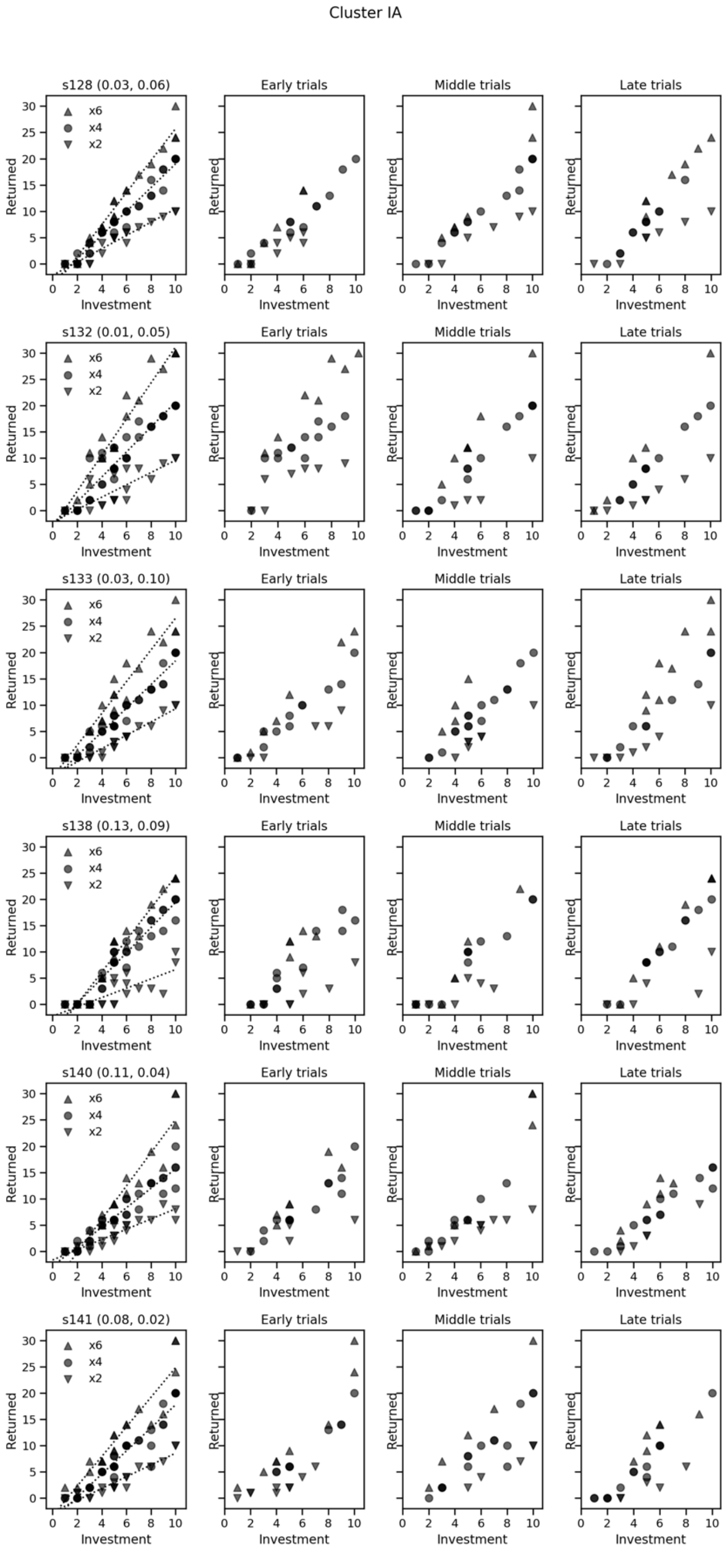

Supplementary fig. 6A, continued

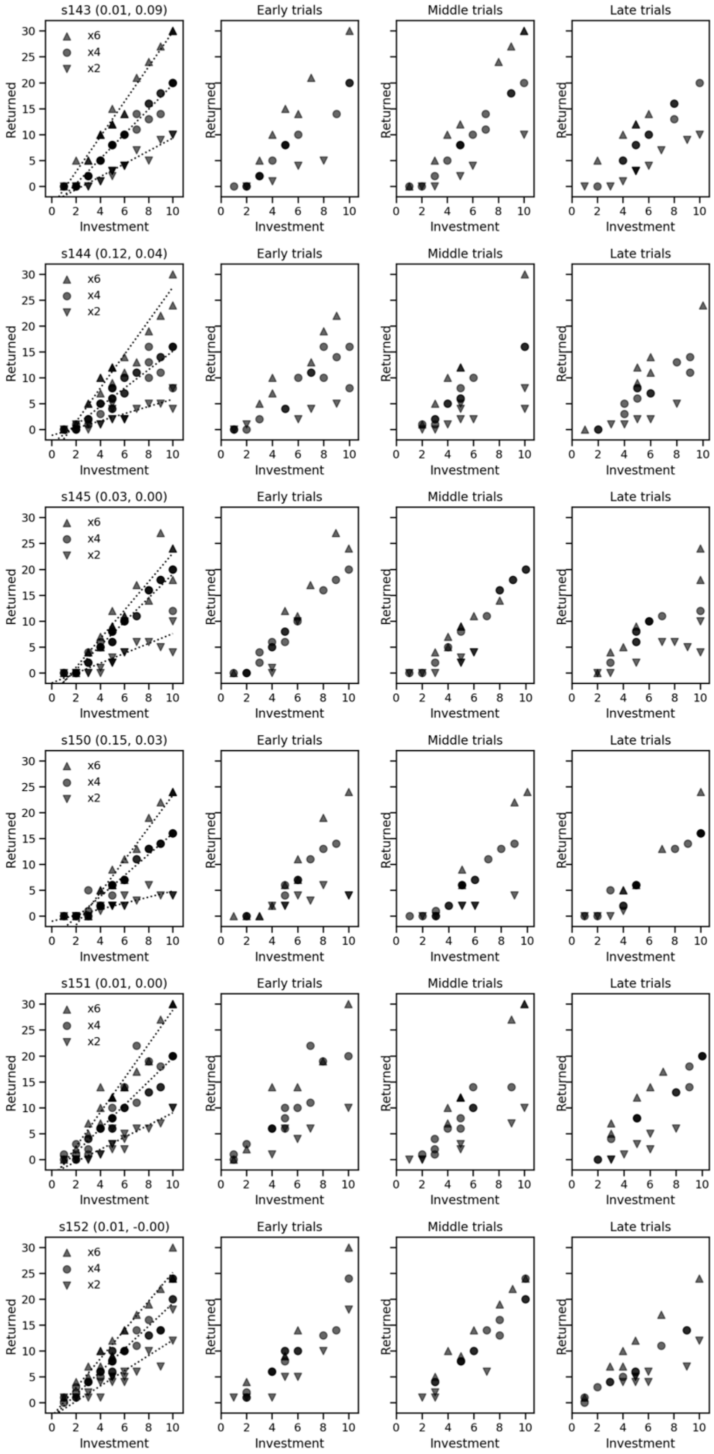

Supplementary fig. 6A, continued

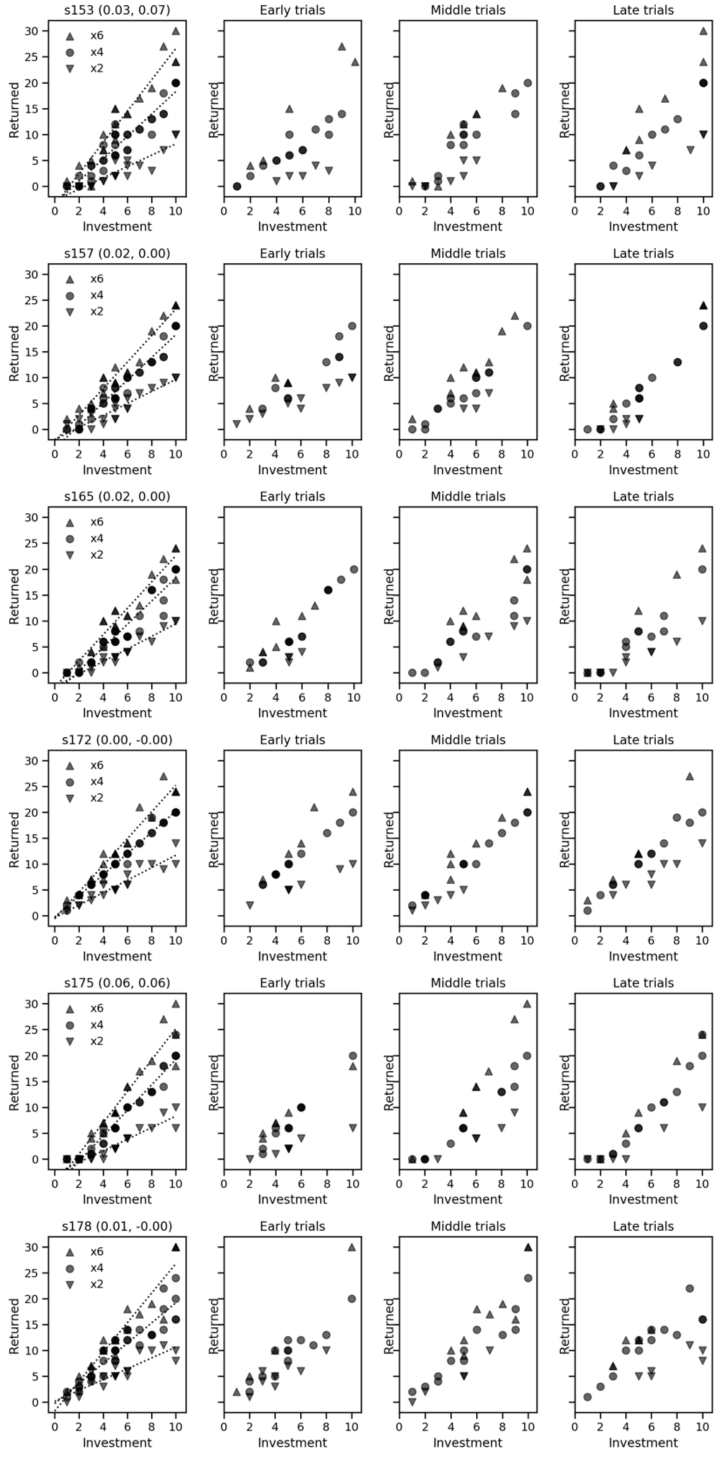

Supplementary fig. 6A, continued

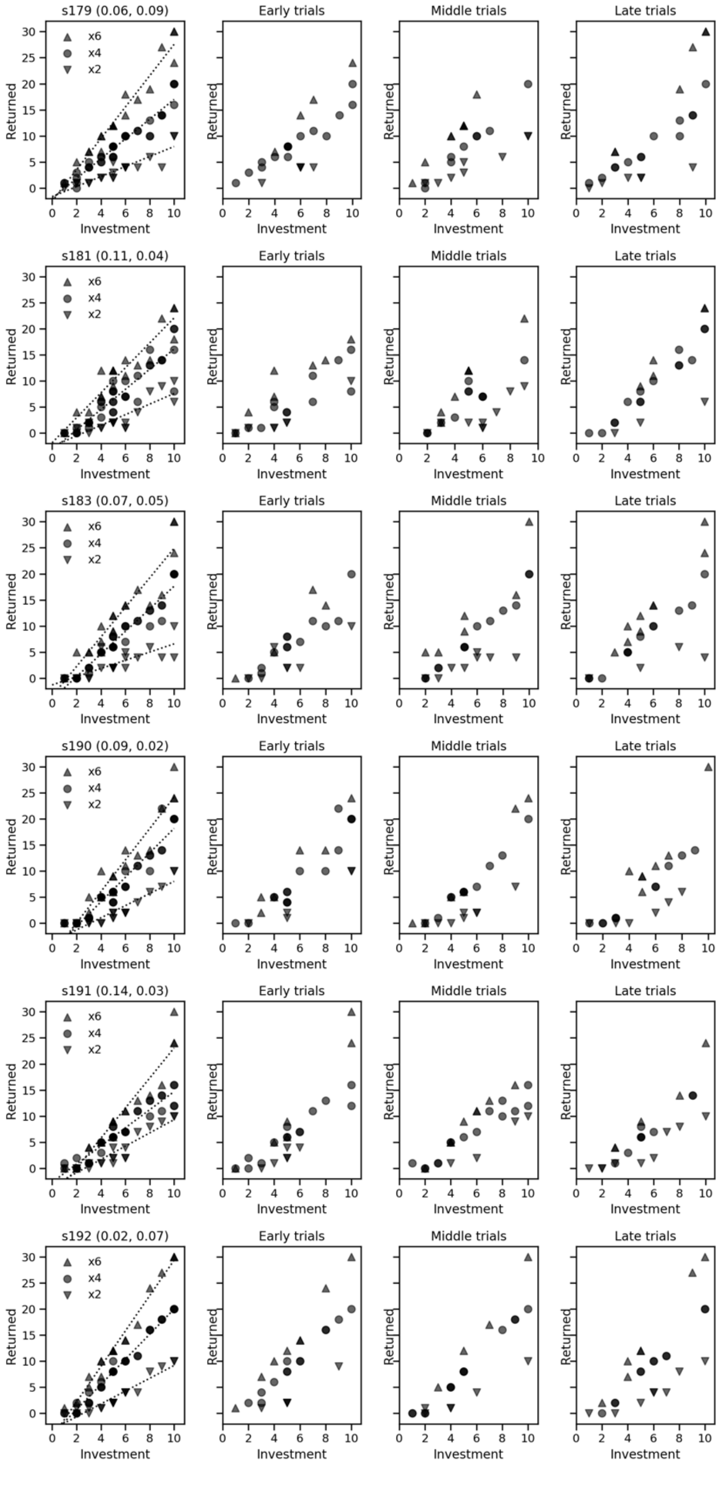

Supplementary fig. 6B

Task behavior over time. Each participant in the guilt-averse (GA) group, split by early, middle, and late trials to illustrate choice consistency over time.

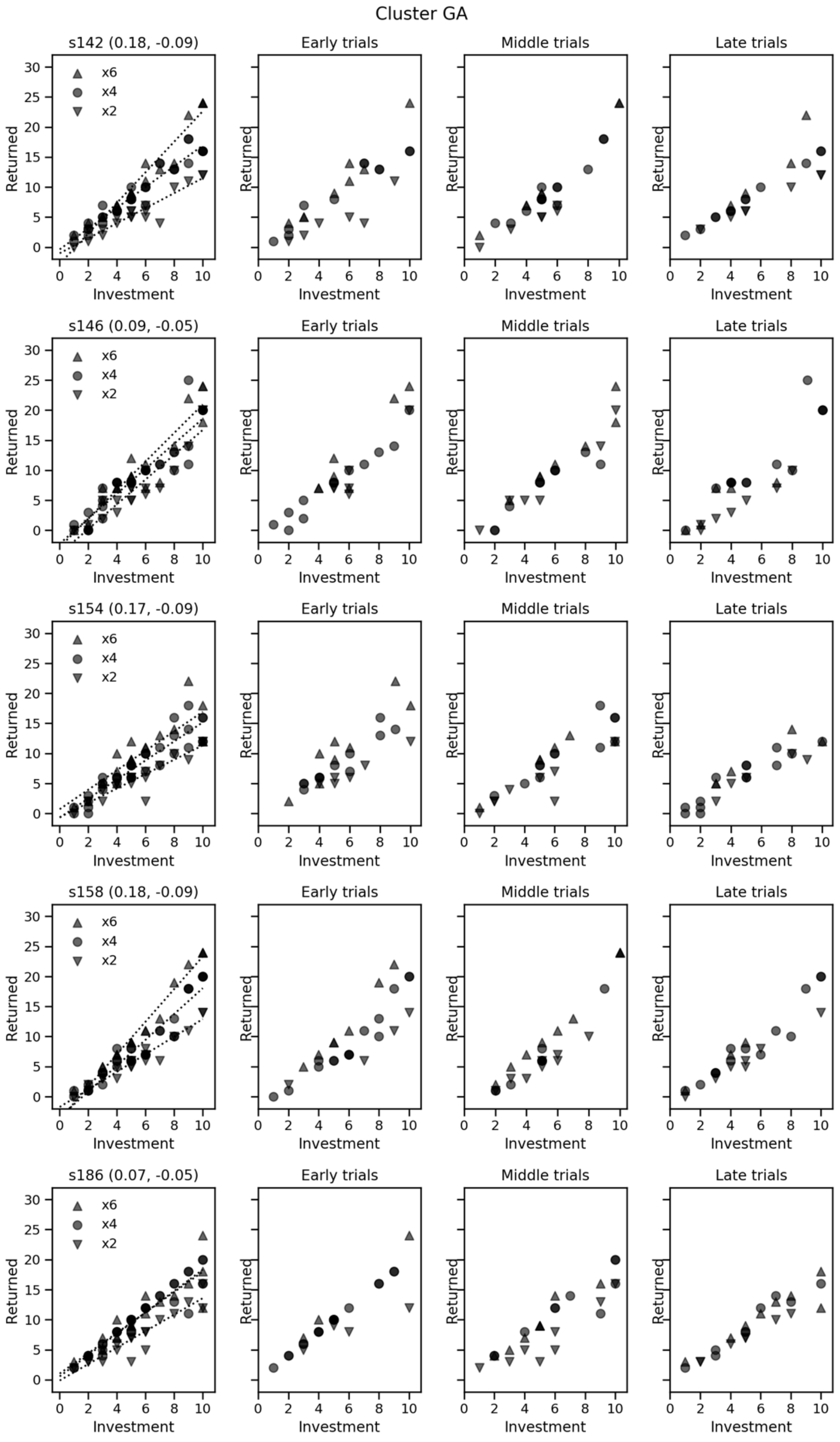

Supplementary fig. 6C

Task behavior over time. Each participant in the greedy (GR) group, split by early, middle, and late trials to illustrate choice consistency over time.

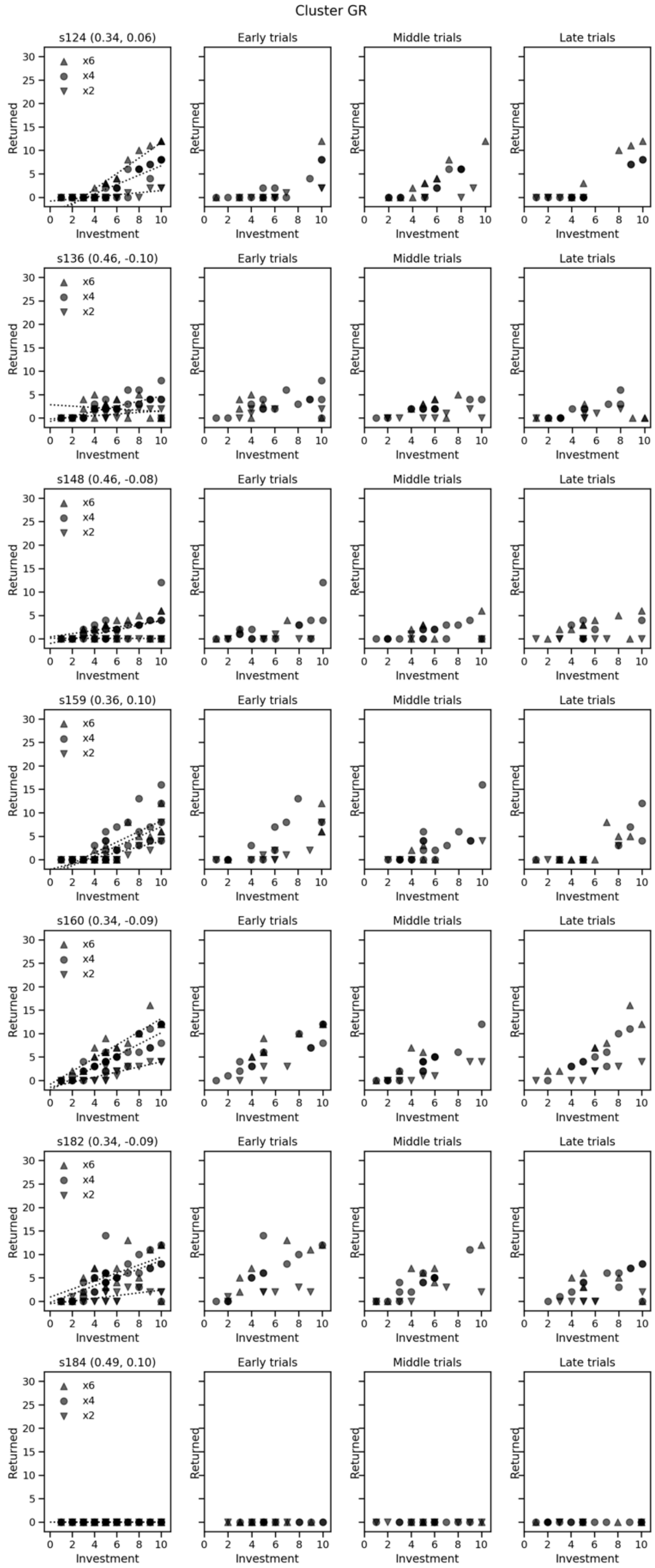

Supplementary fig. 6D

Task behavior over time. Each participant in the morally opportunistic (MO) group, split by early, middle, and late trials to illustrate choice consistency over time.

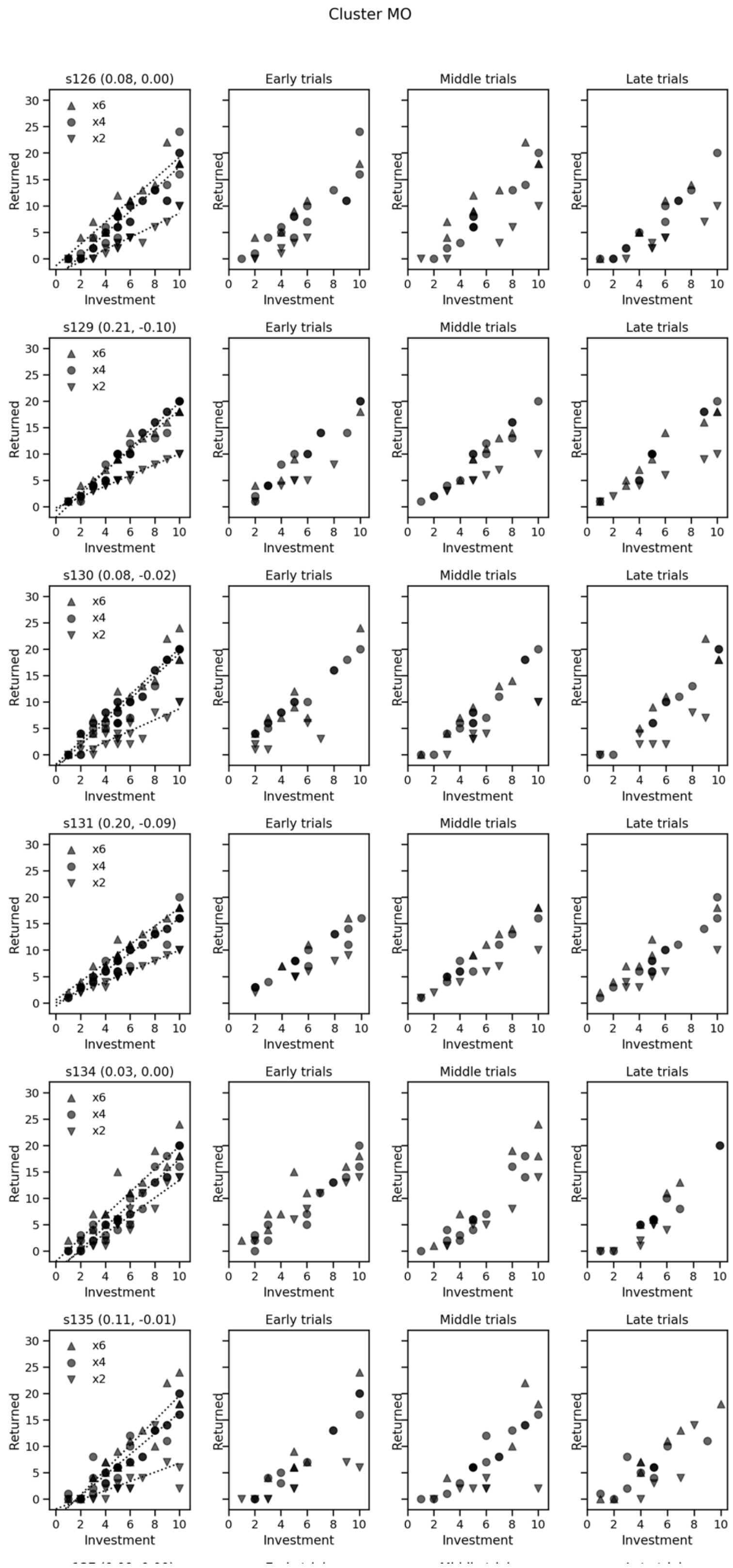

Supplementary fig. 6D, continued

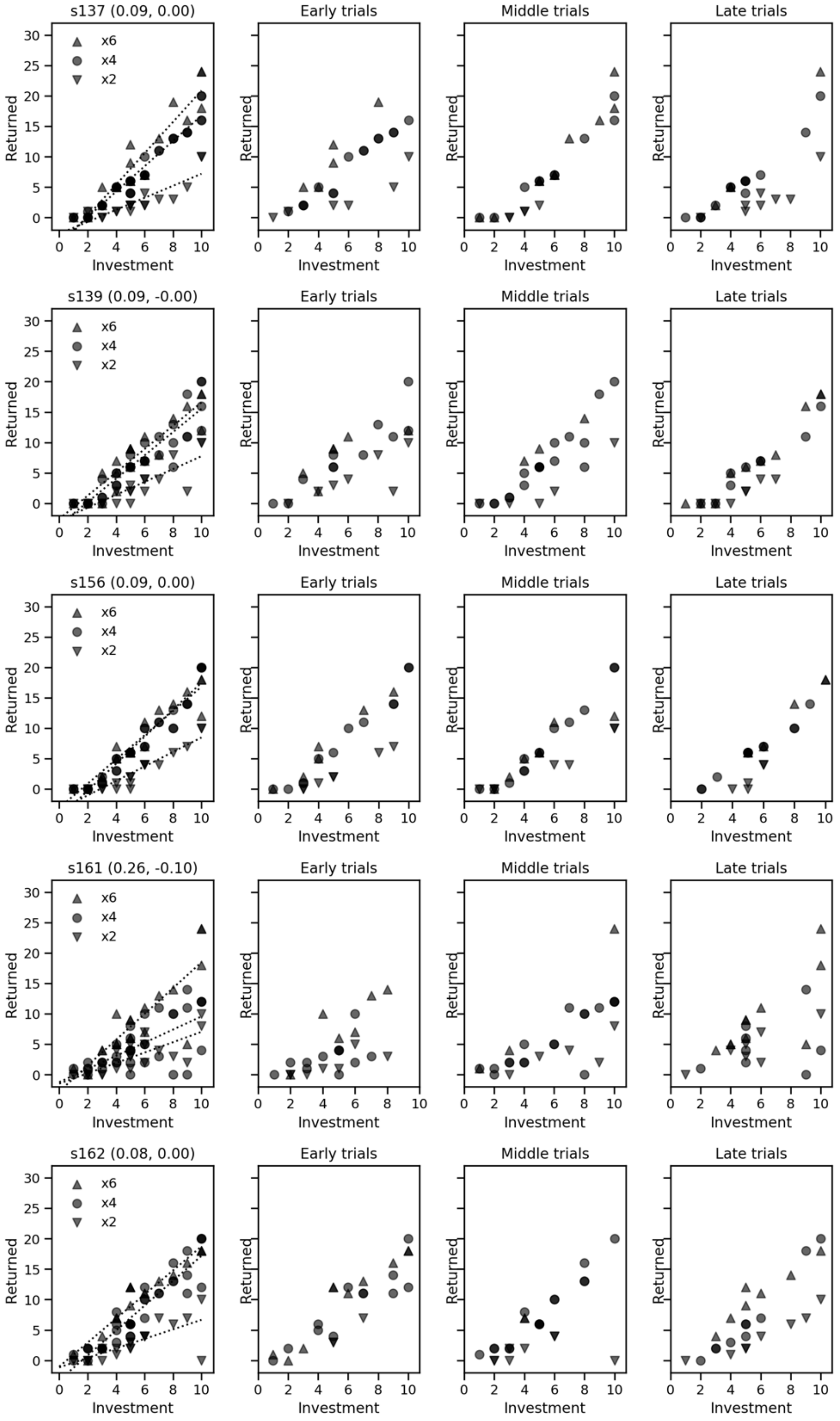

Supplementary fig. 6D, continued

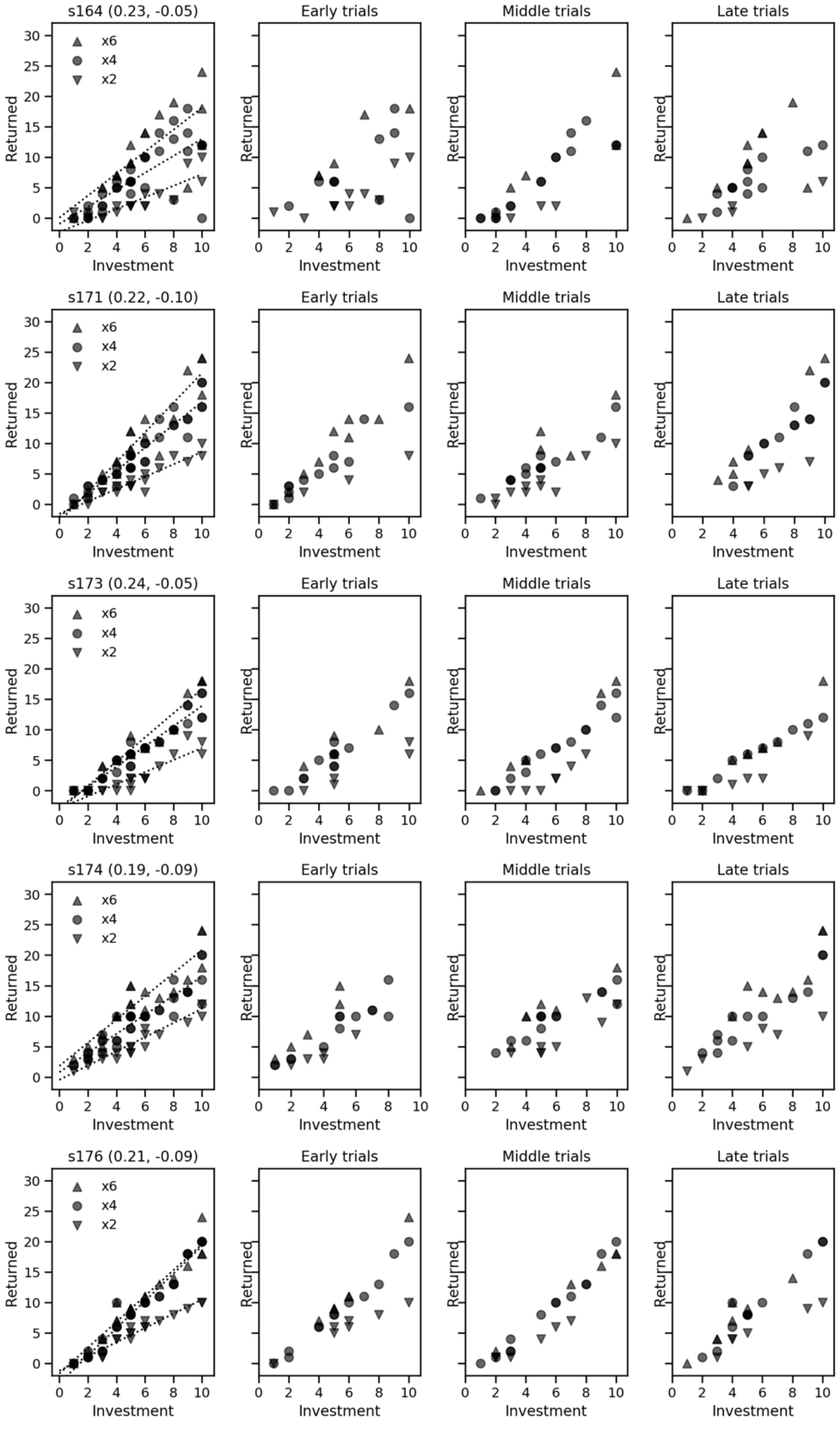

Supplementary fig. 6D, continued

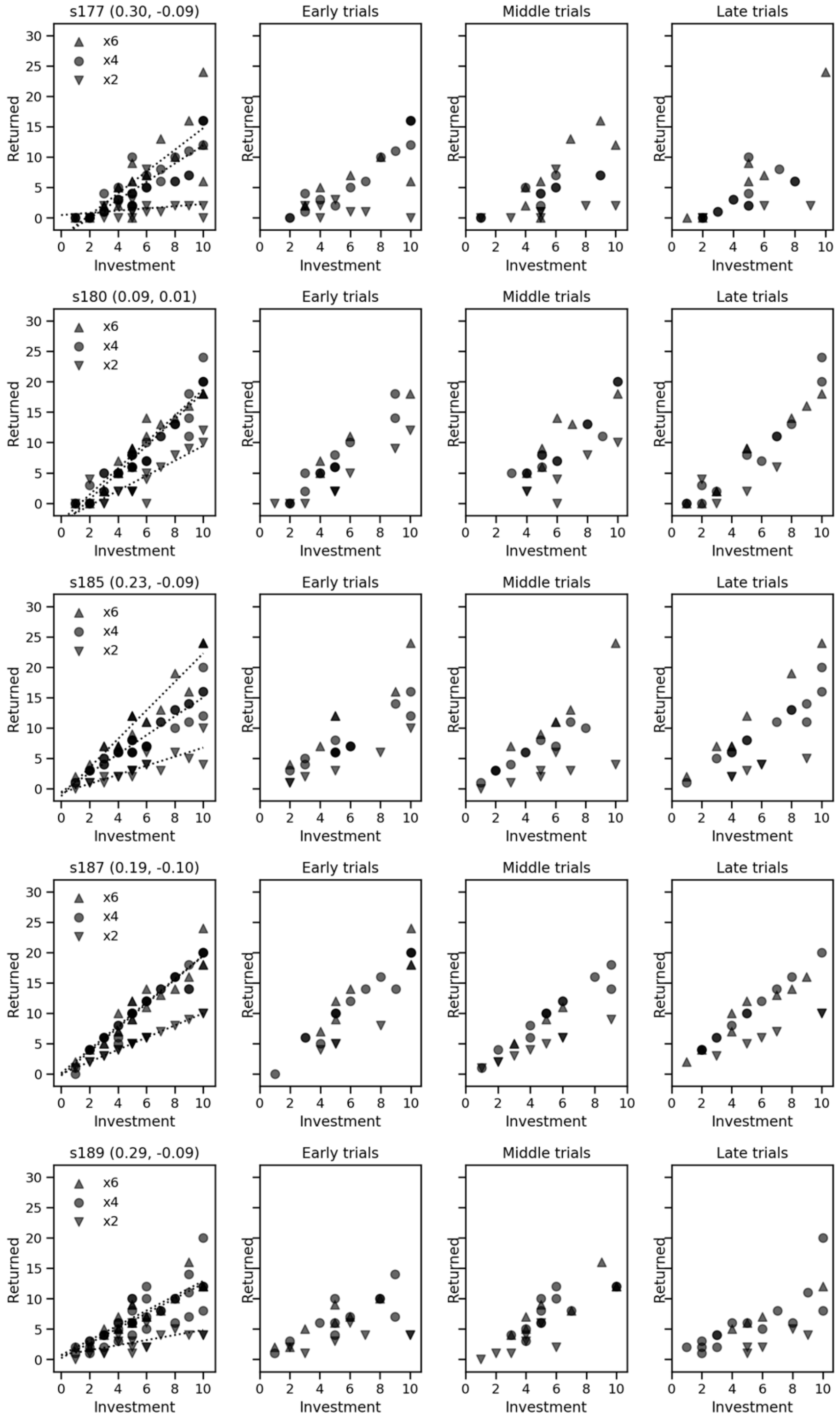

Supplementary Figure 7

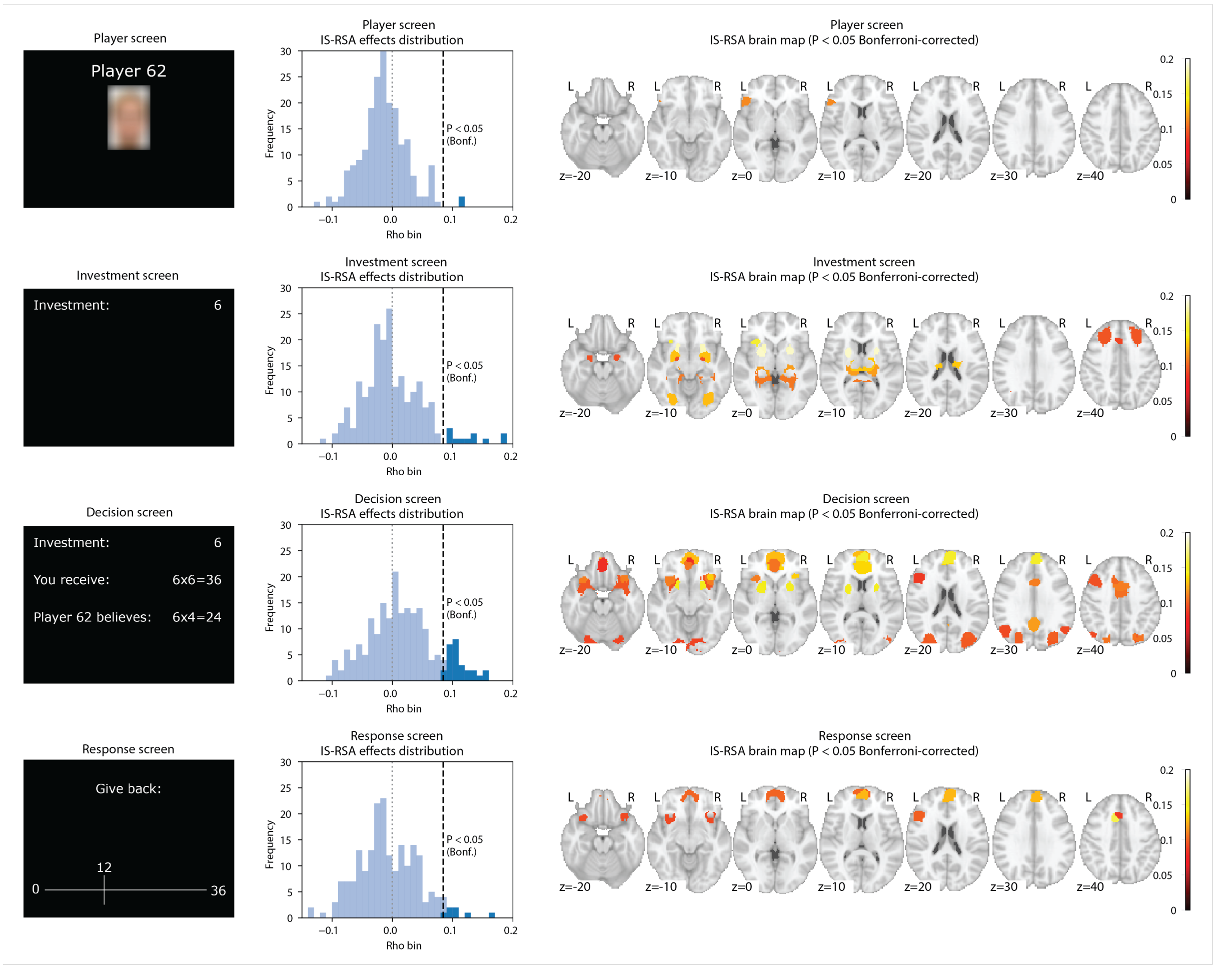

Comparison of inter-subject representational similarity (IS-RSA) effects between the four phases of the HMTG task—the Player, Investment, Decision, and Response screen. IS-RSA effects were by far the most prevalent in the Decision phase of the task, and virtually non-existent when players were introduced to the investor in the Player screen. Left panels: Example screens. Middle panels: Histogram of IS-RSA effects across all 200 brain parcels. IS-RSA effects expressed as Spearman correlation (Rho) between inter-subject distance in computational model parameter space and inter-subject distance in brain parcel representational space, with statistical cutoff at $p < 0.05$ *(Bonferroni-corrected over 200 brain parcels). Right panels: Thresholded brain maps displaying the parcels where a significant positive IS-RSA effect was observed.*

Supplementary Figure 8

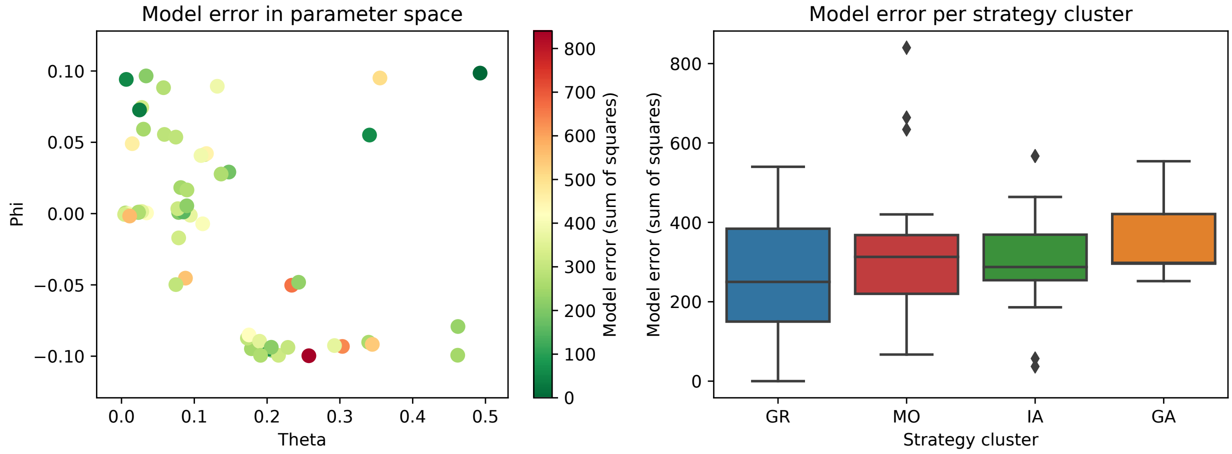

Model error does not depend on moral strategy. Left panel: Model error (sum of squared model residuals per participant) plotted as a function of theta and phi. While participants who correspond to one strategy very strongly generally have a slightly lower model error (e.g. 'extreme' IA or GR participants), model fit is roughly equal for participants throughout the rest of the model parameter space. Of note, participants around the strategy cluster boundaries are fit about equally well by the model as others. Right panel: Box plot of model error distributions in the four different strategy groups. No significant difference was detected between these groups (one-way ANOVA; see Supplementary Table 1).

Supplementary Figure 9

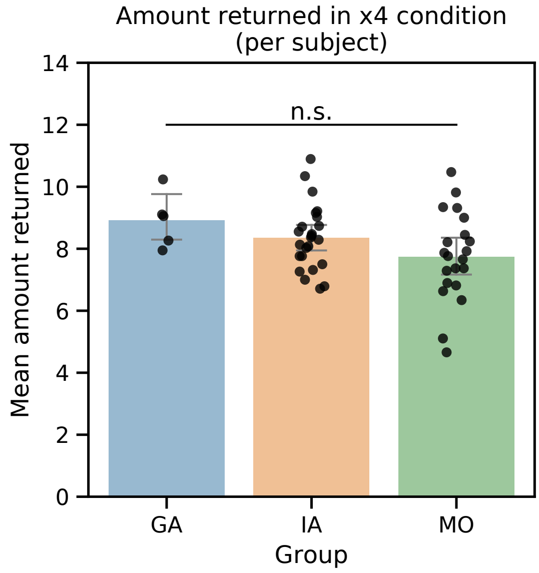

Mean amount returned in the x4 condition per subject, controlled for investment. No significant difference in number of tokens returned was observed between the groups GA, IA, and MO, for whom the computational model predicts the same behavior in this condition (main effect of Group on Amount Returned in linear mixed-effects regression: $F(2,47) = 2.61$*,* $p = 0.084$*).*

Supplementary Figure 10

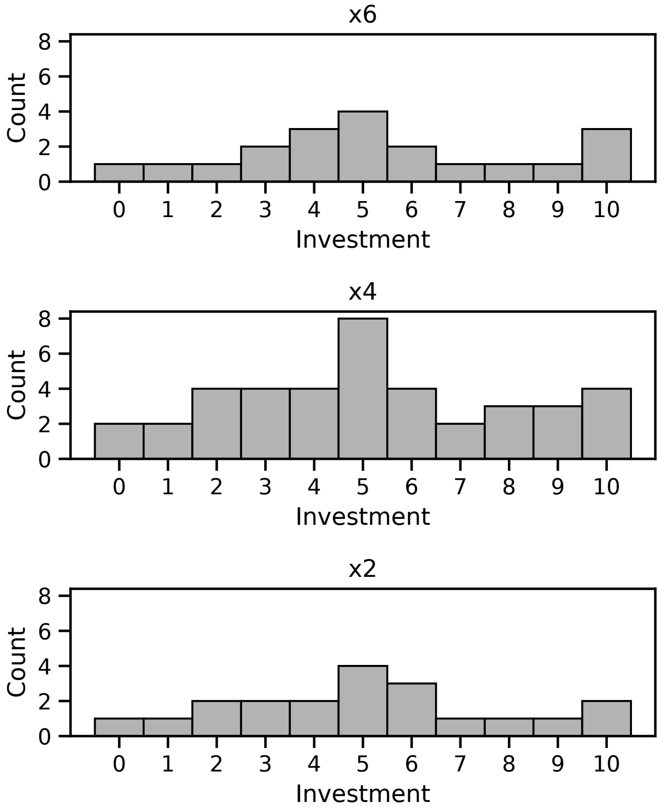

Investment distributions in the three multiplier conditions of the Hidden Multiplier Trust Game.

Supplementary Figure 11

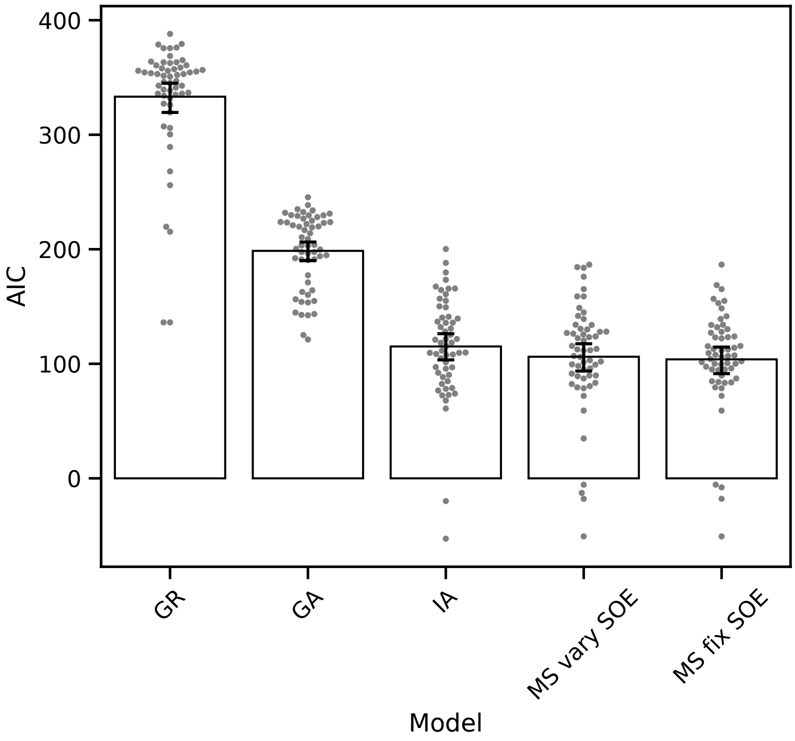

*Model comparisons including the moral strategy model with participant's self-reported second-order expectations as input (MS vary SOE), as well as the moral strategy model with second-order expectations fixed across participants at 50% of 4*Investment (MS fix SOE).*

Supplementary Figure 12

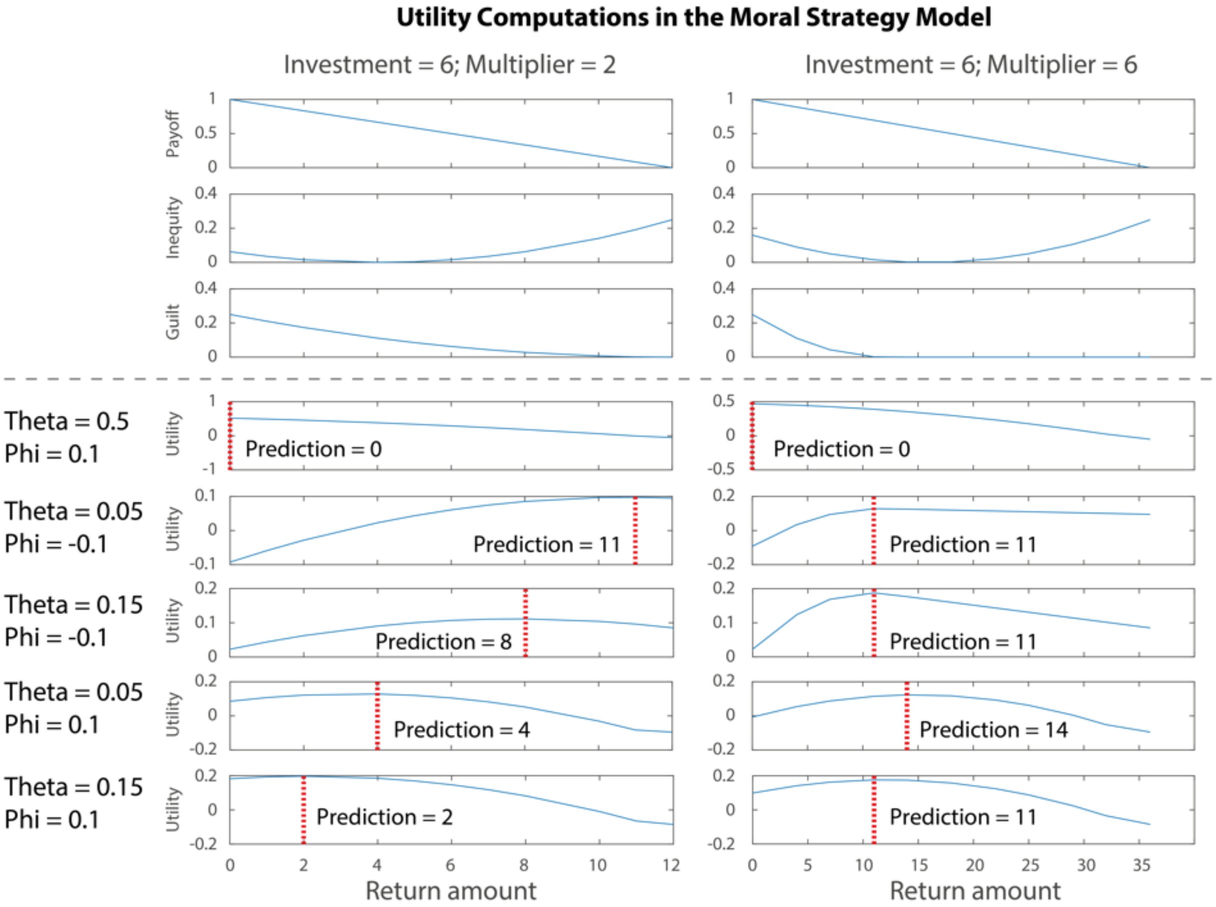

Two examples of the utility curve described by the Moral Strategy Model. Left: investment = 6 and multiplier = 2. Right: investment = 6 and multiplier = 6. Top three panels: the three components of the Moral Strategy Model, i.e. Own share of tokens on this trial (Payoff), Inequity, and Guilt, computed at each possible return amount. Bottom five panels: different combinations of theta and phi correspond to different behavioral predictions ('Prediction', vertical red dotted line). Greed is simulated at theta = 0.5; guilt aversion at theta = 0.05, phi = -0.1; inequity aversion at theta = 0.05, phi = 0.1; other parameter combinations yield intermediate strategies.

Supplementary Figure 13

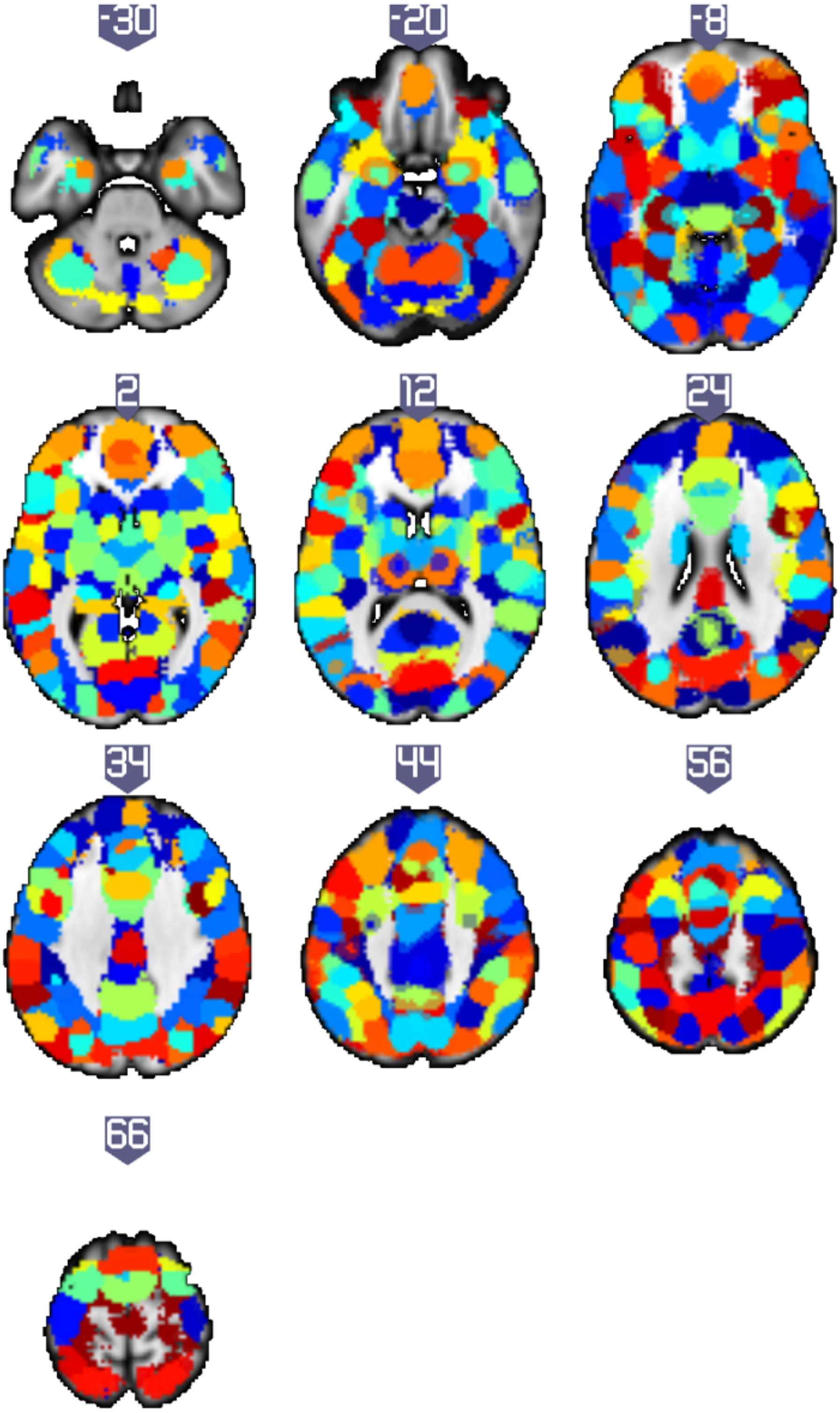

200-parcel parcellation of grey matter in the human brain. Numbers indicate Z coordinate of axial slice in MNI space. This map can be retrieved from http://neurovault.org/images/39711/.

Supplementary Figure 14

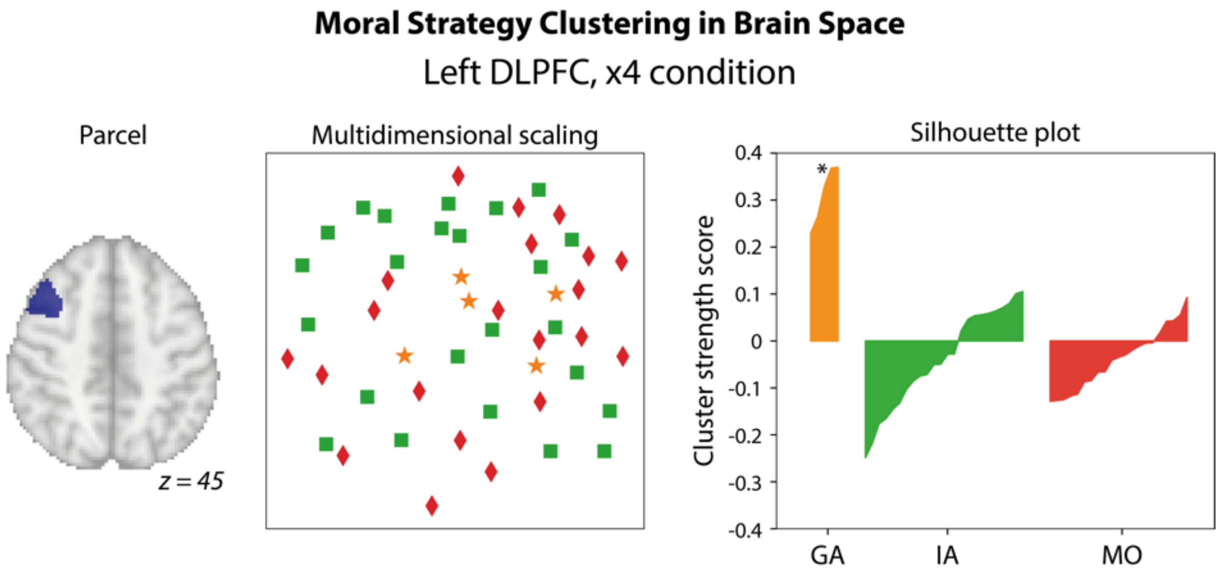

Illustration of the cluster strength metric for the left DLPFC (see left panel) in the x4 condition of the HMTG. Middle panel: multidimensional scaling (MDS) plot of all GA (stars), IA (squares) and MO (diamonds) participants in the 745-dimensional activity pattern space of this parcel. Between-participant distances (i.e. activity pattern differences) are conserved as much as possible by the MDS algorithm in this 2-d representation. Right panel: silhouette plot indicating cluster strength scores for participants in this parcel and condition. In the silhouette plot, each participant is drawn as a (connected) bar indicating their cluster strength score (see Online Methods), and participants are rank-ordered within strategy group . The silhouette plot shows that there is significant pattern clustering for guilt-averse participants in this parcel, which is corroborated by the spatial clustering of GA (but not IA or MO) participants in the MDS plot. Refer to Methods for more details. * $p < 0.05$.
