# Supplementary material for: The computational and neural substrates of moral strategies in social decision-making
Source: Nat Commun. 2019 Apr 2;10:1483. doi: 10.1038/s41467-019-09161-6 (PMC6445121; doi:10.1038/s41467-019-09161-6)
Supplement: Supplementary file 3 — Reporting Summary [file 41467_2019_9161_MOESM3_ESM.pdf]

natureresearch

Corresponding author(s): Jeroen van Baar

Last updated by author(s): Feb 4, 2019

Reporting Summary

Nature Research wishes to improve the reproducibility of the work that we publish. This form provides structure for consistency and transparency in reporting. For further information on Nature Research policies, see Authors & Referees and the Editorial Policy Checklist.

Statistics

For all statistical analyses, confirm that the following items are present in the figure legend, table legend, main text, or Methods section.

n/a	Confirmed	
☐	☒	The exact sample size (*n*) for each experimental group/condition, given as a discrete number and unit of measurement
☐	☒	A statement on whether measurements were taken from distinct samples or whether the same sample was measured repeatedly
☐	☒	The statistical test(s) used AND whether they are one- or two-sided<br>*Only common tests should be described solely by name; describe more complex techniques in the Methods section.*
☒	☐	A description of all covariates tested
☐	☒	A description of any assumptions or corrections, such as tests of normality and adjustment for multiple comparisons
☐	☒	A full description of the statistical parameters including central tendency (e.g. means) or other basic estimates (e.g. regression coefficient) AND variation (e.g. standard deviation) or associated estimates of uncertainty (e.g. confidence intervals)
☐	☒	For null hypothesis testing, the test statistic (e.g. *F*, *t*, *r*) with confidence intervals, effect sizes, degrees of freedom and *P* value noted<br>*Give P values as exact values whenever suitable.*
☒	☐	For Bayesian analysis, information on the choice of priors and Markov chain Monte Carlo settings
☒	☐	For hierarchical and complex designs, identification of the appropriate level for tests and full reporting of outcomes
☐	☒	Estimates of effect sizes (e.g. Cohen's *d*, Pearson's *r*), indicating how they were calculated

Our web collection on statistics for biologists contains articles on many of the points above.

Software and code

Policy information about availability of computer code

Data collection	All stimuli were presented using PsychToolBox 3.0.11 in Matlab 2013a running on Windows PCs.
Data analysis	- Computational models were fit to the behavioral data using the 'least_squares' routine in the Scipy package version 1.1.0 for Python 2.7.15 on Linux;<br>- Clustering of the parameter space of the Moral Phenotype Model was performed using hierarchical clustering implemented in Scipy 1.1.0 for Python 2.7.15 on Linux;<br>- Preprocessing and first-level GLM modeling of fMRI data were performed using SPM12 in Matlab 2014a on Linux;<br>- The inter-subject representational similarity analysis and other similarity analyses were performed using the 'NLTools' package version 0.3.6 (http://github.com/ljchang/nltools) for Python 2.7.15 on Linux;<br>- T-tests were run using Scipy version 1.1.0 for Python 2.7.15 on Linux.<br><br>Custom code was written to call the relevant functions from the above-mentioned packages (for availability, see code availability statement in Methods)

For manuscripts utilizing custom algorithms or software that are central to the research but not yet described in published literature, software must be made available to editors/reviewers. We strongly encourage code deposition in a community repository (e.g. GitHub). See the Nature Research guidelines for submitting code & software for further information.

Data

Policy information about availability of data

All manuscripts must include a data availability statement. This statement should provide the following information, where applicable:

- Accession codes, unique identifiers, or web links for publicly available datasets
- A list of figures that have associated raw data
- A description of any restrictions on data availability

All data and custom code required to reproduce the results in this paper are available from the Donders Institute for Brain, Cognition, and Behavior repository at http://hdl.handle.net/11633/aabwlrrn.

Field-specific reporting

Please select the one below that is the best fit for your research. If you are not sure, read the appropriate sections before making your selection.

☒ Life sciences ☐ Behavioural & social sciences ☐ Ecological, evolutionary & environmental sciences

For a reference copy of the document with all sections, see nature.com/documents/nr-reporting-summary-flat.pdf

Life sciences study design

All studies must disclose on these points even when the disclosure is negative.

Sample size	No sample-size calculation was performed, but the sample size was selected to match previous successful studies using a similar paradigm (e.g. Tricomi, Rangel, Camerer & O'Doherty, Nature, 2010; Chang, Smith, Dufwenberg & Sanfey, Neuron, 2011). Sample size in initial submission was 36. More data was collected based on editorial and reviewer concerns, for a total n of 57 (66 before exclusions).
Data exclusions	The data from nine participants were excluded from the analysis because of:<br>- excessive head movement in the MRI scanner (1) (regular spikes in movement greater than the voxel size);<br>- misunderstanding of the task (2);<br>- disbelief in the task (2);<br>- technical issues (4) (e.g. the scanner-compatible glasses fogged up during the experiment, blocking the participant's vision; the data were not stored properly on the scanner system).<br>All these criteria were pre-established.
Replication	The behavioral findings were reliably reproduced, as described in the manuscript. A replication of the neuroimaging findings was not attempted.
Randomization	The participants in this study were not assigned to experimental groups. Each participant received the same treatment and saw the same stimuli.
Blinding	n/a (see Randomization)

Reporting for specific materials, systems and methods

We require information from authors about some types of materials, experimental systems and methods used in many studies. Here, indicate whether each material, system or method listed is relevant to your study. If you are not sure if a list item applies to your research, read the appropriate section before selecting a response.

Materials & experimental systems

n/a	Involved in the study	
☒	☐	Antibodies
☒	☐	Eukaryotic cell lines
☒	☐	Palaeontology
☒	☐	Animals and other organisms
☐	☒	Human research participants
☒	☐	Clinical data

Methods

n/a	Involved in the study	
☒	☐	ChIP-seq
☒	☐	Flow cytometry
☐	☒	MRI-based neuroimaging

Human research participants

Policy information about studies involving human research participants

Population characteristics	All participants: mean age = 21.3 ± 2.1 years, 39 women and 18 men. Students of economics were excluded from participation, as they were potentially familiar with game theory or the Trust Game. All participants were screened for significant health or

	neurological problems and had normal or corrected-to-normal vision.
Recruitment	Participants were recruited from the Nijmegen student population using an online research participation tool.
Ethics oversight	CMO Arnhem-Nijmegen, The Netherlands

Note that full information on the approval of the study protocol must also be provided in the manuscript.

Magnetic resonance imaging

Experimental design

Design type	Task; event-related design
Design specifications	80 trials per subject, divided over two runs of 40 trials each. Variable inter-trial interval between 2 and 4 seconds (uniformly distributed).
Behavioral performance measures	For each trial, all button presses with which the participant moved the response slider were recorded, including their timing relative to the start of the response screen. The final position of the slider in the response screen was recorded as the participant's response for that trial. We checked whether all participants actually moved the slider to make sure that they completed the task as expected.

Acquisition

Imaging type(s)	T1-weighted structural imaging to enable normalization of scans to common space; T2*-weighted functional imaging to measure BOLD response.
Field strength	3.0
Sequence & imaging parameters	Structural imaging: 192 sagittal slices were acquired using an MPRAGE sequence (TR 2300 ms, voxel size 1x1x1 mm). Functional imaging: thirty-five ascending slices were acquired (3.0 mm slice thickness; 0.5 mm slice gap; 3.5x3.5x3.0 mm voxel size) using a multi-echo pulse sequence (224 mm field of view (FOV); 64 x 64 matrix; 90° flip angle; 2250 ms repetition time (TR); echo times (TE) 9.4 ms, 20.6 ms, 32 ms, and 43 ms).
Area of acquisition	Whole brain except cerebellum

Diffusion MRI ☐ Used ☒ Not used

Preprocessing

Preprocessing software	fMRI data pre-processing was carried out using SPM12 in Matlab version 2014a. Pre-processing of the functional images consisted of slice time correction to the middle slice, coregistration to the T1-weighted anatomical scan, normalization to MNI-space, and smoothing with a Gaussian kernel of 8 mm full width at half maximum.
Normalization	Data were normalized using the forward deformation fields obtained by segmenting the anatomical scan using the Segment routine in SPM12. Trilinear interpolation was used to normalize the functional images; 4th-degree B-spline interpolation for the structural images.
Normalization template	We normalized all images to MNI space using the ICBM152 template (default in SPM12)
Noise and artifact removal	The motion parameters obtained during realignment were stored and added to the GLM analysis as nuisance regressors.
Volume censoring	n/a

Statistical modeling & inference

Model type and settings	For first-level analysis, a GLM was constructed for each participant using boxcar regressors for each task condition. All four screens of the task were taken as conditions, with the trials in the decision and response screens split by multiplier level. A parametric modulator for investment size was added during the investment screen. The two runs were modelled by separate regressors in the same GLM. To account for residual variance, the temporal derivative of each condition regressor was added to the model as well as a constant regressor for each entire run. The resulting GLM was convolved with SPM's canonical hemodynamic response function. The model was corrected for temporal autocorrelations using a first-order autoregressive model and a standard high-pass filter (cut-off at 128 s) was used to exclude low-frequency drifts.<br><br>We used the GLM beta maps for the decision screen (x2, x4 and x6 condition) for the subsequent inter-subject RSA and similarity analyses.
Effect(s) tested	For the IS-RSA, we averaged the GLM beta maps for the x4 condition trials of the decision screen per participant (i.e. over the two runs), to obtain one decision activity map per participant. At each of 200 parcels of the brain (see 12. Analysis), we then computed the correlation distance between the activity maps of each pair of participants. We calculated the rank correlation of the Euclidean distance between each pair of participants in the parameter space of

the computational model to the correlation distance of each pair in the brain distance space. We used permutation testing with 10,000 samples to assess whether the observed Spearman correlation was significantly greater than zero, Bonferroni-corrected for the number of parcels (i.e. $p < 0.05/200$, i.e. $p < 0.00025$).

For the cluster strength analysis, we again used permutation testing: we permuted the sign of the observed cluster strength scores within a moral phenotype group 10,000 times and compared the actual mean cluster strength in the moral phenotype group to the distribution of the permuted scores.

For the analysis of moral opportunism similarity to inequity and guilt aversion, we used t-tests to assess the effect of condition (x6 versus x2) on similarity of an MO subject's activity map to the GA-IA z-difference map.

Specify type of analysis: ☐ Whole brain ☐ ROI-based ☒ Both

Anatomical location(s): We used a whole-brain parcellation based on meta-analytic functional co-activation of the Neurosynth database (parcellation available at http://neurovault.org/images/39711).

Statistic type for inference (See Eklund et al. 2016): No mass univariate analyses were used in this paper.

Correction: We used permutation tests wherever possible, and Bonferroni-corrected our statistics for repetition over 200 parcels (whole brain).

Models & analysis

n/a	Involved in the study
☒	☐ Functional and/or effective connectivity
☒	☐ Graph analysis
☒	☐ Multivariate modeling or predictive analysis
